# Organizing pneumonia in mice and men

**DOI:** 10.1186/s12967-016-0933-6

**Published:** 2016-06-10

**Authors:** Nicole Izykowski, Mark Kuehnel, Kais Hussein, Kristin Mitschke, Michael Gunn, Sabina Janciauskiene, Axel Haverich, Gregor Warnecke, Florian Laenger, Ulrich Maus, Danny Jonigk

**Affiliations:** Institute of Pathology, Hannover Medical School, Carl-Neuberg-Straße 1, 30625 Hannover, Germany; Department of Immunology, Duke University Medical Center, Durham, NC USA; Department of Experimental Pneumology, Hannover Medical School, Hannover, Germany; Department of Thoracic Surgery, Hannover Medical School, Hannover, Germany; Biomedical Research in Endstage and Obstructive Lung Disease Hannover (BREATH), Hannover, Germany; German Center for Lung Research (Deutsches Zentrum für Lungenforschung, DZL), Bad Nauheim, Germany

**Keywords:** Organizing pneumonia, CCL2 transgenic mouse, Laser-assisted microdissection

## Abstract

**Background:**

Organizing pneumonia is a reaction pattern and an inflammatory response to acute lung injuries, and is characterized by intraluminal plugs of granulation tissue in distal airspaces. In contrast to other fibrotic pulmonary diseases, organizing pneumonia is generally responsive to corticosteroids. However, some patients do not respond to treatment, leading to respiratory failure and potentially death (up to 15 % of patients). In order to devise new therapeutic strategies, a better understanding of the disease’s pathomechanisms is warranted. We previously generated a mouse model overexpressing CCL2, which generates organizing pneumonia-like changes, morphologically comparable to human patients. In this study, we investigated whether the histopathological similarities of human and murine pulmonary organizing pneumonia lesions also involve similar molecular pathways.

**Methods:**

We analyzed the similarities and differences of fibrosis-associated gene expression in individual compartments from patients with organizing pneumonia and transgenic (CCL2) mice using laser-assisted microdissection, real-time PCR and immunohistochemistry.

**Results:**

Gene expression profiling of human and murine organizing pneumonia lesions showed in part comparable expression levels of pivotal genes, notably of TGFB1/Tgfb1, TIMP1/Timp1, TIMP2/Timp2, COL3A1/Col3a1, CXCL12/Cxcl12, MMP2/Mmp2 and IL6/Il6. Hence, the transgenic CCL2 mouse model shows not only pathogenomic and morphological features of human organizing pneumonia but also a similar inflammatory profile.

**Conclusions:**

We suggest that the CCL2-overexpressing transgenic mouse model (CCL2 Tg mice) is suitable for further investigation of fibrotic pulmonary remodeling, particularly of organizing pneumonia pathogenesis and for the search for novel therapeutic strategies.

## Background

Organizing pneumonia (OP) is a reaction pattern and an inflammatory response towards acute lung injuries. Originally, OP was described by Davison and coworkers in 1983 and referred to as “bronchiolitis obliterans organizing pneumonia” (BOOP) [1]. Nowadays, in order to avoid confusion with the distinct airway disease bronchiolitis obliterans (BO), the term OP is preferred.

In principle, there are two manifestations of OP: (1) cryptogenic organizing pneumonia (COP) of unknown cause [2] and (2) secondary organizing pneumonia (SOP), based on a known cause, e.g. infections (bacterial, viral, fungal or parasitic), drugs (antibiotics, antiepileptics, immunomodulators), inflammatory diseases like connective tissue disease, vasculitis or following lung/bone marrow transplantation [3]. In general, OP is considered to be a non-specific, aberrant reparative response to a multitude of injurious stimuli.

Histomorphologically, OP is characterized by intraluminal plugs of granulation tissue (predominantly within the alveolar ducts and surrounding alveoli), with whorls of fibroblasts and activated myofibroblasts embedded in a connective matrix (so-called Masson bodies) [3]. In contrast to other fibrotic lung diseases, like BO, OP is generally responsive to corticosteroids [4, 5] and the majority of patients with COP respond to this common therapy with rapid clinical, radiological and functional improvement. However, up to 15 % of patients do not respond to treatment at all and show a progressive deterioration, leading to respiratory failure and, potentially, death [3, 5, 6]. Recognition of the underlying pathological mechanisms of OP, especially of its progressive variant, might help to identify those patients who will progress to respiratory failure [7]. Cell culture models are not suitable to recapitulate the complex remodeling mechanisms of OP. Therefore, animal models depicting human OP are needed to develop new therapeutic strategies. Among the animal models available, at least two limitations can be identified: firstly, they are dedicated to study airway obliteration (OAR) rather than BOOP—or more precisely OP—and secondly, they do not depict either disease satisfactorily [8].

In a previous study, we observed that transgenic mice overexpressing human CC chemokine ligand 2 (hCCL2) under control of the surfactant protein C promoter in type II alveolar epithelial cells (CCL2 Tg mice) responded to infectious challenge with *Streptococcus pneumoniae* (*S. pneumoniae*) with profound OP-like lesions involving the peripheral bronchioles and alveoli, particularly in the later resolving phase of bacterial pneumonia (day 7 post-infection), therefore recapitulating OP manifestations morphologically comparable to those seen in human patients [9].

The initial trigger leading to OP is thought to be a circumscribed epithelial injury, which induces the death of pneumocytes and the formation of gaps in the basal lamina [3, 4]. Fibroblasts and inflammatory cells like lymphocytes, neutrophils and eosinophils then infiltrate the alveolar interstitium and form fibroinflammatory buds. This results in the formation of granulation tissue, which then progresses into a network of polypoid plugs within the lumen of bronchioles, alveolar ducts and adjacent peribronchiolar alveoli [3–5]. The cells involved express proinflammatory factors which in turn attract more leukocytes and profibrotic progenitor cells and lead to deposition and remodeling of more extracellular matrix (ECM) [10]. In human lungs, leukocytes and stroma cells within OP lesions express proinflammatory genes (e.g. interleukin 6/IL6), pivotal profibrotic signaling factors such as transforming growth factor-beta 1 (TGFB1) and downstream Sma and Mad-related protein family members (SMADs) as well as profibrotic/antiangiogenic matricellular thrombospondin 1 (THBS1). Expression of these factors is generally associated with collagen production within pulmonary fibrotic lesions [10, 11]. Accumulation and persistence of collagen in the ECM are dependent on the expression of bone morphogenetic proteins (BMPs), matrix metalloproteinases (MMPs) and complementary tissue inhibitors of MMPs (TIMPs). Inflammatory cells also express protein tyrosine kinase 2 (PTK2), a cytoplasmic factor which contributes to the focal adhesions which form between cells and the adjacent ECM [10, 11]. Furthermore, stroma cell-secreted chemokine (C-X-C motif) ligand 12 (CXCL12) and its leukocyte-derived chemokine (C-X-C motif) receptor 4 (CXCR4) are overexpressed, which indicates mobilization of CXCL12/CXCR4-mediated leukocytes [10, 11].

In the present study, we investigated whether the histopathological pulmonary OP lesions in humans and mice share a similar, OP-related molecular phenotype, which in turn would identify CCL2 transgenic mice as a suitable humanized mouse model of OP, allowing future evaluation of OP-directed therapeutic strategies. Hence, we analyzed fibrosis-associated gene expression profiles from individual compartments of human and transgenic CCL2 mouse lungs.

## Methods

### Human specimens and study groups

OP represents a reaction pattern leading? Towards a variety of lung injuries, which manifests with a distinct and common morphology despite different underlying causes. Therefore, we investigated formalin-fixed and paraffin-embedded (FFPE) lung samples with organizing pneumonia (n = 5) from explanted native lungs of different clinical backgrounds, harvested during allogeneic lung transplantation and downsizing samples (n = 6) from donor lungs, which were resected just before lung transplantation, as controls. The average age of patients (two women and three men) with OP at the time of transplantation was 42.4 years (range 5.3–70.2 years). FFPE samples were retrieved from the archives of the Institute of Pathology, Hannover Medical School (Hannover, Germany) and were handled anonymously according to the requirements of the local ethics committee (MHH ethics committee vote no. 2050-2013).

### Human CCL2 transgenic mice

The murine FFPE lung tissue was obtained from the archives of the Department of Experimental Pneumology (Hannover Medical School). The generation of the human CCL2 expressing mice was previously described [12]. Briefly cDNA encoding human MCP-I was inserted into the previously described SRF2T plasmid. Targeted expression of a dominant negative FGF receptor blocks branching morphogenesis and epithelial differentiation of the mouse lung and microinjected into fertilized eggs [13].

Adult mice derived from these eggs were infected with a Pneumolysin-producing clinical isolate of the *S. pneumoniae as* described in [12]. Both study groups comprised 8–12-week-old Balb/c mice. Briefly, a clinical isolate of *S. pneumoniae* capsular group 19 strain EF3030 was cultivated in Todd-Hewitt broth (Difco) supplemented with 0.1 % yeast extract to mid-log phase. Aliquots of bacterial suspensions were snap-frozen in liquid nitrogen and stored at −80 °C until further use. Bacterial numbers were quantified by plating of serial dilutions on sheep blood agar plates (BD Biosciences) followed by incubation of the plates at 37 °C/5 % CO_2_ for 18 h and subsequent determination of CFU.

Infection of CCL2 overexpressing and wild-type mice with *S. pneumoniae* was done using freshly prepared dilutions of thawed aliquots adjusted to ∼3 × 10^7^ CFU/mouse. Briefly, tracheas were exposed by surgical resection, and intratracheal instillation of the pneumococci was performed under stereomicroscopic control (MS 5; Leica) using a 26-gauge catheter (Abbocath) inserted into the trachea. After instillation, the neck wound was closed with sterile sutures as described in [12].

At 7 days post infection Wild-type and CCL2 overexpressing mice were killed via an overdose of Isoflurane. Subsequently, lungs were inflated in situ with a prewarmed solution of Tissue-Tek (Sakura) kept at 37 °C. Thereafter, the lungs were carefully removed and immersed in PBS-buffered formaldehyde solution (4.5 %, pH 7.0) for at least 24 h fixation at room temperature. Lung tissue samples were paraffin-embedded, and lung sections of 5 μm were stained with H&E and Elastica-van-Gieson and examined histopathologically using a Zeiss Axiovert 200 M microscope.

The histopathological evaluation of transgenic mice overexpressing human CCL2 (n = 10) and corresponding wild-type control mice (n = 10) was performed by an experienced lung pathologist (DJ) on H&E and Elastica van Gieson-stained sections. Notably, transgenic mice expressed CCL2 under the control of the surfactant protein C promoter. Therefore, human CCL2 is selectively expressed by type II alveolar epithelial cells, resulting in hCCL2-dependent alveolar accumulation of monocyte and lymphocyte subsets under baseline conditions, as characterized previously in detail [9, 12].

### Laser-assisted microdissection and RNA extraction

FFPE tissue sections of 5 µm thickness were mounted on a poly-l-lysine-coated membrane fixed onto a metal frame for laser-microdissection (MMI Molecular Machines Industries AG, Glattbrugg, Switzerland). After deparaffinization and hemalaun staining, non-obliterated bronchioles and OP lesions from human and murine lung tissue were isolated with the CellCut Plus system (MMI). The microdissected material was suspended directly into the adhesive cap with proteinase K buffer, incubated overnight and RNA was isolated with phenol/chloroform extraction according to our established procedure [14].

### cDNA generation and real-time PCR of CCL2 and fibrosis-associated genes

The total amount of extracted RNA was converted to cDNA by using the High Capacity cDNA Reverse Transcription Kit (Applied Biosystems, Foster City, CA, USA), following the manufacturer’s protocol. This cDNA was then preamplified with nonrandom PCR primers and PreAmp MasterMix (Applied Biosystems, Carlsbad, CA, USA) which results in a decrease of CT values by 14 PCR cycles [15]. For the subsequent quantitative real-time PCR (TaqMan 7500 Real-Time PCR system, Applied Biosystems, Carlsbad, CA, USA), the preamplified cDNA was mixed with TaqMan Gene Expression Master Mix and the individual TaqMan Gene Expression Assay. For verification of CCL2 overexpression, transcripts of human CCL2 as well as murine endogenous Ccl2 were evaluated.

A panel of fibrosis- and inflammation-associated murine and human genes selected on the basis of previous work was analyzed: Il6/IL6, Tgfb1/TGFB1, Thbs1/THBS1, Smad1/SMAD1 and Smad3/SMAD3, Bmp4/BMP4, bone morphogenetic protein receptor type-1b (Bmpr1b/BMPR1B), collagen type III, alpha 1 (Col3a1/COL3A1), Mmp2/MMP2, Timp1/TIMP1 and Timp2/TIMP2, Ptk2/PTK2, Cxcl12/CXCL12 and Cxcr4/CXCR4 (Table 1) [10, 11]. The gene DNA-directed RNA polymerase II subunit RPB1 (Polr2a/POLR2A) was used as an endogenous reference gene. For negative controls, cDNA was replaced by water.

**Table 1:** Description of inflammation- and fibrosis-associated genes investigated in human (OP and control) lungs and in transgenic and nontransgenic mice

Gene name	Approved symbol (human/mouse)	Approved symbol (mouse)	Function
Bone morphogenetic protein 4	BMP4	Bmp4	BMPs are a group of growth factors also known as cytokines and are part of the transforming growth factor beta superfamily. BMPs induce cartilage and bone formation
Bone morphogenetic protein receptor, type 1B	BMPR1B	Bmpr1b	On ligand binding, a receptor complex forms consisting of two type II and two type I transmembrane serine/threonine kinases. Type II receptors phosphorylate and activate type I receptors which autophosphorylate, then bind and activate SMAD transcriptional regulators
Chemokine (C-C motif) ligand 2	CCL2	Ccl2	This chemokine is a member of the CC subfamily which is characterized by two adjacent cysteine residues. This cytokine displays chemotactic activity for monocytes and basophils but not for neutrophils or eosinophils. It has been implicated in the pathogenesis of diseases characterized by monocytic infiltrates, like psoriasis, rheumatoid arthritis and atherosclerosis. It binds to chemokine receptors CCR2 and CCR4
Collagen, type III, alpha 1	COL3A1	Col3a1	Collagen type III occurs in most soft connective tissues (skin, lung, uterus, intestine and the vascular system) along with type I collagen. Involved in the regulation of cortical development
Chemokine (C-X-C motif) ligand 12	CXCL12	Cxcl12	Chemoattractant active on T-lymphocytes, monocytes, but not neutrophils. It functions as the ligand for the G protein-coupled receptor, chemokine (C-X-C motif) receptor 4, and plays a role in many diverse cellular functions, including embryogenesis, immune surveillance, inflammation response, tissue homeostasis, and tumor growth and metastasis
Chemokine (C-X-C motif) receptor 4	CXCR4	Cxcr4	Receptor for the C-X-C chemokine CXCL12/SDF-1 that transduces a signal by increasing intracellular calcium ion levels and enhancing MAPK1/MAPK3 activation. Acts as a receptor for extracellular ubiquitin; leading to enhanced intracellular calcium ions and reduced cellular cAMP levels
Interleukin 6	IL6	Il6	Cytokine that functions in inflammation and the maturation of B cells. In addition, it has been shown to be an endogenous pyrogen capable of inducing fever in people with autoimmune diseases or infections. The protein is primarily produced at sites of acute and chronic inflammation, where it is secreted into the serum and induces a transcriptional inflammatory response through interleukin 6 receptor alpha
Matrix metallopeptidase 2	MMP2	Mmp2	Ubiquitinous metalloproteinase that is involved in diverse functions such as remodeling of the vasculature, angiogenesis, tissue repair, tumor invasion, inflammation, and atherosclerotic plaque rupture. As well as degrading extracellular matrix proteins, it can also act on several non-matrix proteins such as big endothelin-1 and beta-type CGRP promoting vasoconstriction
Protein tyrosine kinase 2	PTK2	Ptk2	Non-receptor protein-tyrosine kinase that plays an essential role in regulating cell migration, adhesion, spreading, reorganization of the actin cytoskeleton, formation and disassembly of focal adhesions and cell protrusions, cell cycle progression, cell proliferation and apoptosis
SMAD family member 1	SMAD1	Smad1	This protein mediates the signals of the bone morphogenetic proteins (BMPs), which are involved in a range of biological activities including cell growth, apoptosis, morphogenesis, development and immune responses. In response to BMP ligands, this protein can be phosphorylated and activated by the BMP receptor kinase, type I. The phosphorylated form of this protein forms a complex with SMAD4, which is important for its function in the transcription regulation
SMAD family member 3	SMAD3	Smad3	Receptor-regulated SMAD (R-SMAD) that is an intracellular signal transducer and transcriptional modulator activated by TGF-beta (transforming growth factor) and is thought to play a role in the regulation of carcinogenesis
Transforming growth factor, beta 1	TGFB1	Tgfb1	Multifunctional protein that controls proliferation, differentiation and other functions in many cell types. Many cells synthesize TGFB1 and have specific receptors for it. It positively and negatively regulates many other growth factors. At low concentrations in concert with IL-6 and IL-21, it leads to expression of the IL-17 and IL-23 receptors, favoring differentiation to Th17 cells
Thrombospondin 1	THBS1	Thbs1	This protein is an adhesive glycoprotein that mediates cell-to-cell and cell-to-matrix interactions. It can bind to fibrinogen, fibronectin, laminin, type V collagen and integrins alpha-V/beta-1 and has been shown to play roles in platelet aggregation, angiogenesis, and tumorigenesis
Tissue inhibitor of metalloproteinase 1	TIMP1	Timp1	Metalloproteinase inhibitor that functions by forming one to one complexes with target metalloproteinases, such as collagenases, and irreversibly inactivates them by binding to their catalytic zinc cofactor. Acts on all MMPs, except on MMP14. Also functions as a growth factor that regulates cell differentiation, migration and cell death and activates cellular signaling cascades
Tissue inhibitor of metalloproteinase 2	TIMP2	Timp2	In addition to an inhibitory role against metalloproteinases, this protein has a unique role among TIMP family members in its ability to directly suppress the proliferation of endothelial cells. As a result, the encoded protein may be critical to the maintenance of tissue homeostasis by suppressing the proliferation of quiescent tissues in response to angiogenic factors, and by inhibiting protease activity in tissues undergoing remodeling of the extracellular matrix

Information is taken from HUGO gene Nomenclature Committee, Uniprot and NCBI-Gene

### Immunohistochemistry

Serially cut sections (1 µm) were deparaffinized and rehydrated with descending alcohol concentrations and then immunohistochemically stained for different inflammation- and fibrosis-associated targets using commercially available antibodies (Table 2) and a standard ABC protocol [10, 11].

**Table 2:** Antibodies, source and dilutions used for immunohistochemistry

Antibody	Company	Dilution
BMP4	Abcam	1:750
BMPR1B	Abnova	1:500
CCL2	Acris Antibodies	1:100
COL3A1	Acris Antibodies	1:50
CXCL12	Acris Antibodies	1:100–200
IL-6	Abcam	1:500
MMP2	Abcam	1:200
TGFB1	Abcam	1:100
TIMP1	Zytomed/Emergo	1:500
TIMP2	Abcam	1:50

### Statistics

CT values were calculated by normalization to the mean expression of the reference gene (Polr2a/POLR2A) and converted into 2^−ΔCT^ values with Microsoft office Excel 2010 (Microsoft, Redmond, WA, USA). The statistical analysis was performed with GraphPad Prism 5.0 (GraphPad Software, San Diego, CA, USA) using the Mann–Whitney U test for comparison of wild-type and transgenic mice as well as the human OP lesions and normal airways from controls, and the Wilcoxon signed-rank test to compare the respective compartment/lesions within the transgenic mice. Statistically significant differences between the different groups were assumed when p values were <0.05.

## Results

### Analysis of CCL2/Ccl2 expression levels in human und murine lung tissues

**Fig. 1:**
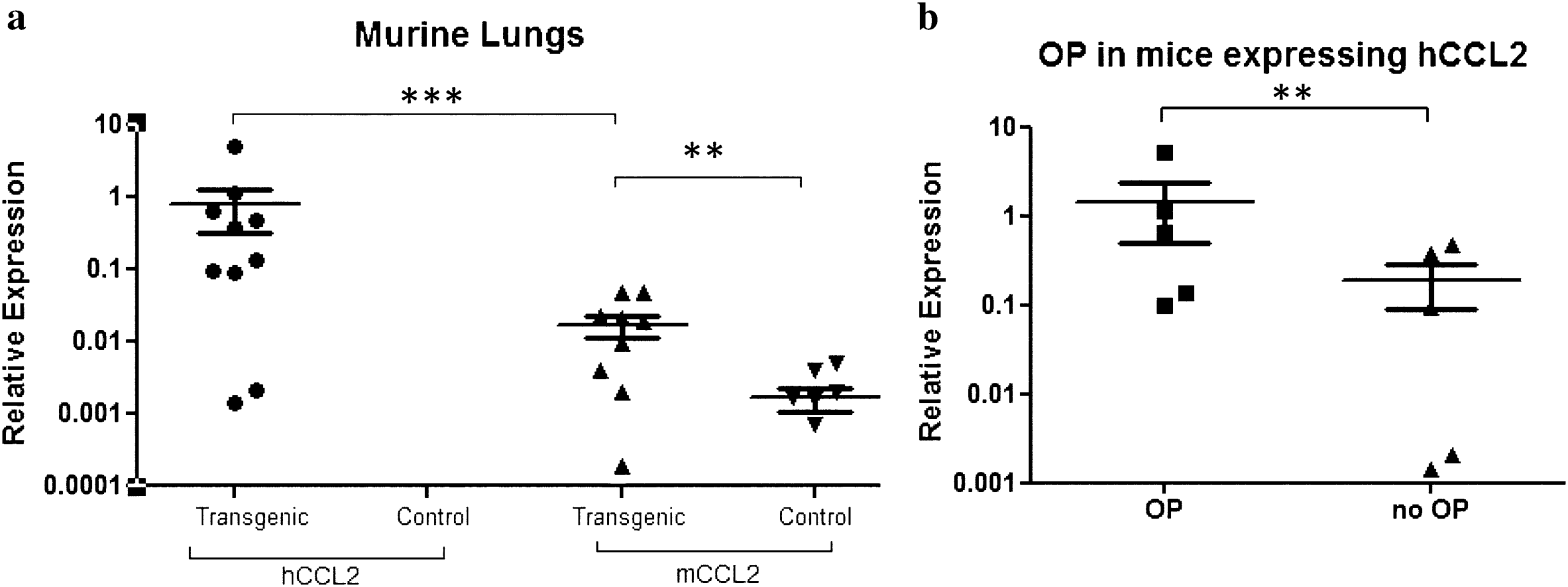
**a** mRNA expression of human CCL2 (hCCL2) versus murine Ccl2 (mCCL2) in murine lungs. Human CCL2 is only expressed in transgenic mice, but not in controls. The murine (endogenous) Ccl2 was expressed in both transgenic and nontransgenic animals. Log scale (*y*-axis). **b** Transgenic animals expressing hCCL2 divided into those with and without OP manifestation

Gene expression levels of CCL2/Ccl2 were analyzed via quantitative PCR. As expected, CCL2 tg mice expressed human CCL2 in their lungs parenchyma, while the control group was negative. Moreover, CCL2 Tg mice expressed higher levels of human CCL2 than endogenous murine Ccl2 (Fig. 1a), while the development of OP correlated with the expression level in CCL2 Tg mice (Fig. 1b). In human lungs, the endogenous CCL2 was not significantly different in samples from OP lungs compared to healthy controls (data not shown).

### Histopathological manifestation of OP in transgenic mice

**Fig. 2:**
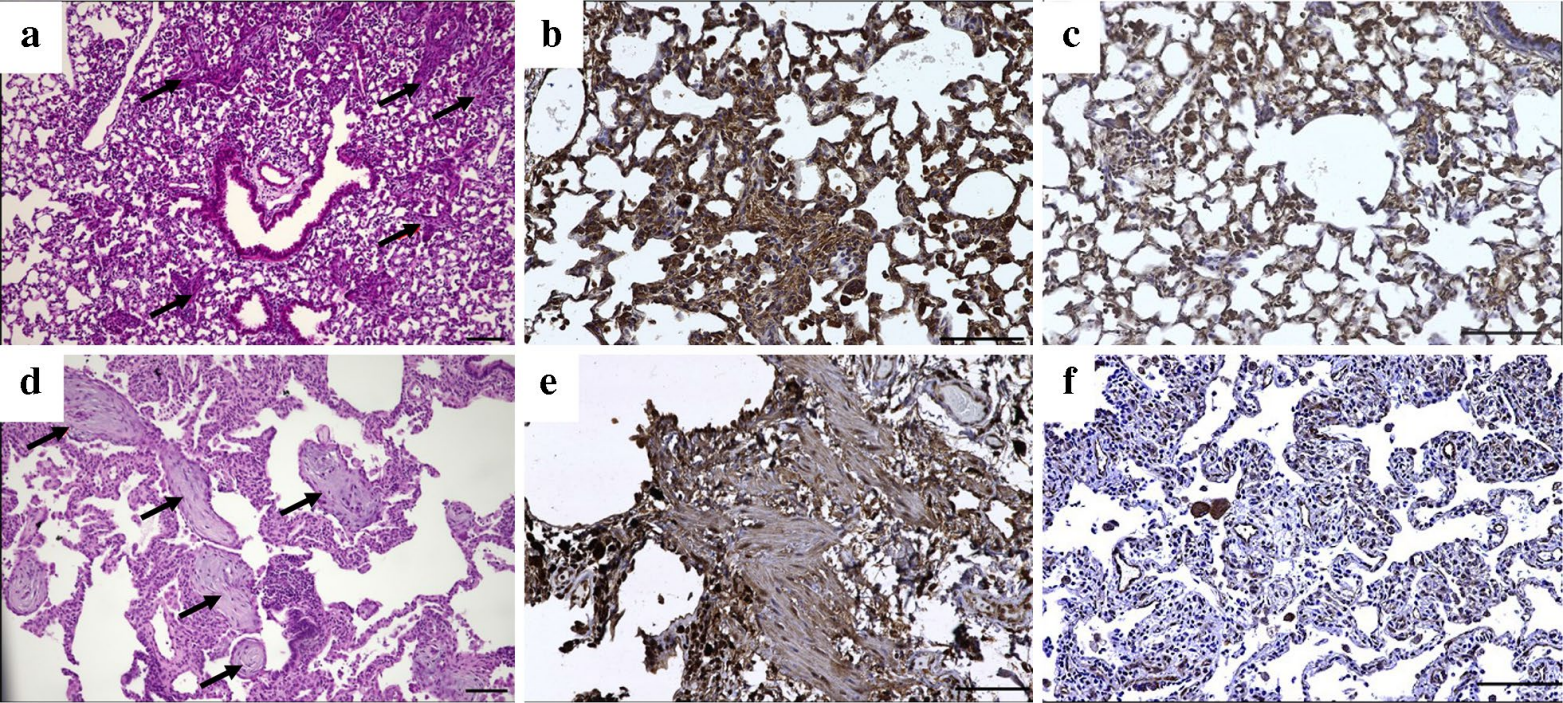
OP lesions stained by hematoxylin and eosin (**a**, **d**), and immunohistochemically against MMP2 (**b**, **e**) and IL6 (**c**, **f**) in transgenic mice (**a**–**c**) and in human OP lungs (**d**–**f**). Histopathologically, the organizing pneumonia pattern (*arrows*), which is characterized by excessive intraalveolar proliferations of granulation tissue, associated with lymphocytic pneumonitis in the surrounding lung tissue, is comparable in humans and mice. *Scale bars* correspond to 100 µm

Consistent with previous reports [9], histopathological evaluation of archived lung tissues from CCL2 Tg and WT mice showed that 50 % of CCL2 Tg mice developed OP after infection with *S. pneumoniae*, while control mice did not respond with OP after infection with *S. pneumoniae* (Fig. 2). However, it turned out that animals that developed OP show a higher expression of hCCL2 compared to animals without OP lesions (Fig. 1b).

### Deregulation of inflammation-associated genes in OP lesions

Via isolation of OP lesions and non-affected airways using laser-microdissection and complementary immunohistochemistry, gene expression profiles were analyzed in a compartment-specific manner.

**Fig. 3:**
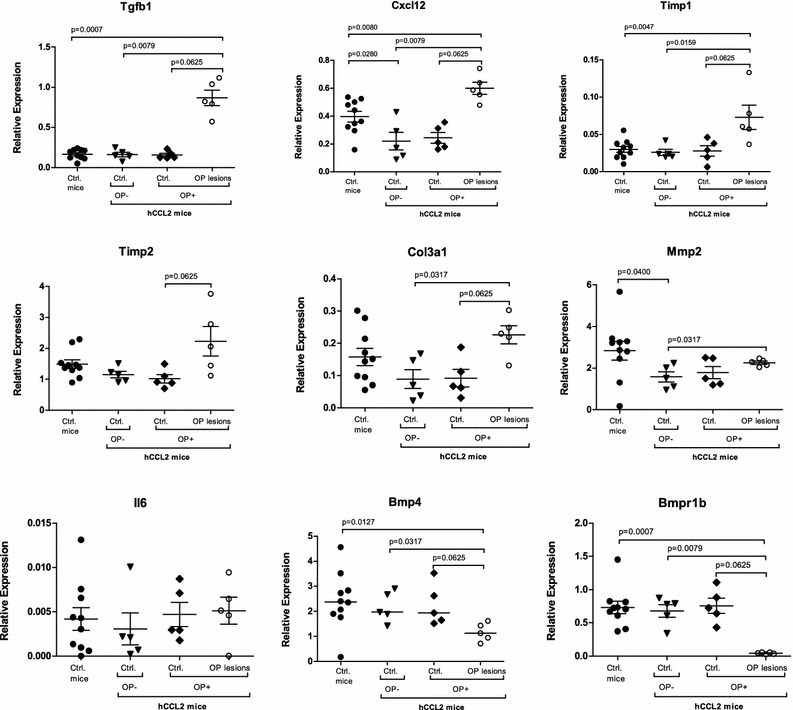
Gene expression levels of the Tgfb1/TGFB1 cascade in murine OP lesions, compared to controls. TGFB1/Tgfb1, CXCL12/Cxcl12, TIMP1/Timp1, TIMP2/Timp2, COL3A1/Col3a1 and MMP2/Mmp2 show an OP lesion-specific overexpression in both human and murine lungs. In contrast, BMP4 and BMPR1B were upregulated in human OP lesions, compared to controls, while in murine lungs both genes were downregulated. There was no significant difference in the expression level of IL6/Il6. Ctrl.—control(s)

**Fig. 4:**
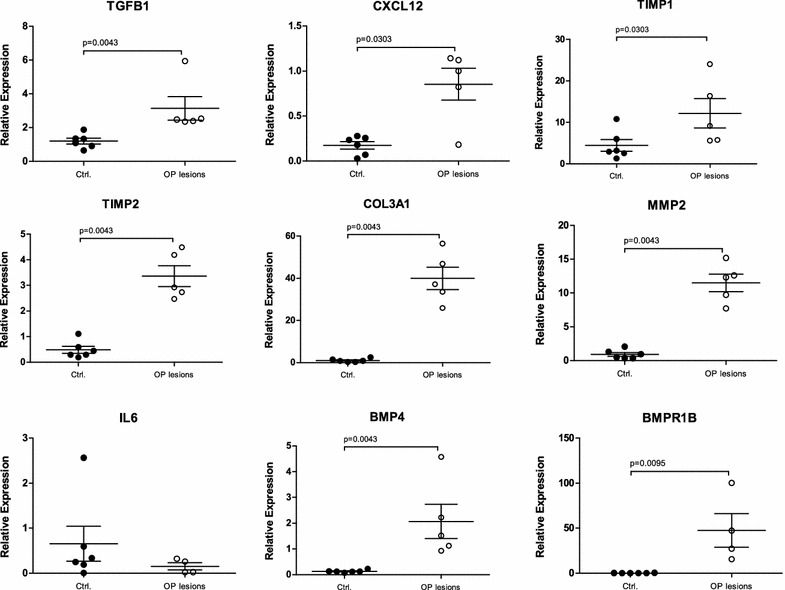
Gene expression levels of the Tgfb1/TGFB1 cascade in human OP lesions, compared to controls. TGFB1/Tgfb1, CXCL12/Cxcl12, TIMP1/Timp1, TIMP2/Timp2, COL3A1/Col3a1 and MMP2/Mmp2 show an OP lesion-specific overexpression in both human and murine lungs. In contrast, BMP4 and BMPR1B were upregulated in human OP lesions, compared to controls, while in murine lungs both genes were downregulated. There was no significant difference in the expression level of IL6/Il6. Ctrl.—control(s)

CCL2 transgene expression was not directly linked to expression of inflammatory mediators. Both, CCL2 Tg and WT mice exhibited similar levels of transcript expression of almost all factors under investigation in non-remodeled pulmonary compartments. However, an OP lesion-specific overexpression was found for Tgfb1, Cxcr4, Cxcl12, Thbs1, Timp1, Timp2 and Col3a1 (Figs. 3, 4), while Bmp4 and its complementary receptor Bmpr1b were downregulated. Levels of Mmp2 and Ptk2 showed a trend towards higher levels in OP, but differences were only significant at the level of intra-individual comparison to non-remodeled lung tissue in CCL2 transgenic mice. Smad1, Smad3 and Il6 were expressed at similar levels in CCL2 Tg and control mice and were not deregulated in OP lesions.

Although the overall expression levels in human lung tissue were in total higher than in murine lungs, laser-microdissected OP lesions and non-remodeled bronchioles displayed comparable levels of significance regarding fibrosis- and inflammation-associated genes. Genes like TGFB1, TIMP1, TIMP2, COL3A1, CXCL12 and MMP2 were significantly upregulated in OP lesions from human lung explant tissue (Figs. 3, 4), while BMP4 and BMPR1B showed contrary results between OP lesions from human and mouse lungs. In murine OP lesions, these two genes were downregulated, whereas human OP lesions demonstrated increased expression levels compared to controls. THBS1, CXCR4, PTK2 and IL6 were expressed at similar levels in human OP lesions and controls. While SMAD1 and SMAD3 were not upregulated in transgenic and control animals, SMAD1 showed an elevated expression level in human OP lesions, whereas SMAD3 was downregulated (Figs. 3, 4). Immunohistochemistry confirmed the mRNA expression results as summarized in Fig. 2 and Table 3.

**Table 3:**
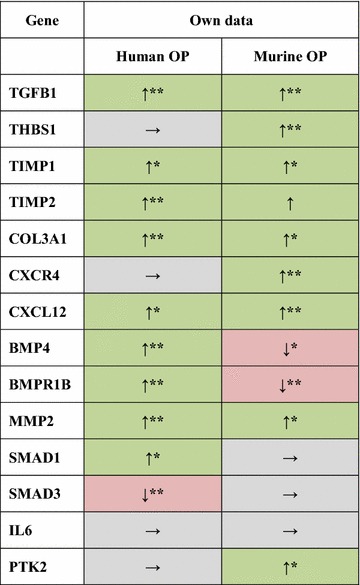
Comparison of expression levels of inflammation- and fibrosis-associated genes in human and murine OP lesions

*OP* organizing pneumonia, ↑ upregulation (green) in OP lesions compared to non-remodeled bronchioles of hCCL2 mice without OP manifestation (OP-Ctrl.)/human controls, → no significant differences in gene expression (gray), ↓ downregulation (red) compared to non-remodeled bronchioles of hCCL2 mice without OP manifestation (OP-Ctrl.)/human controls

* p ≤ 0.05; ** p ≤ 0.01; *** p ≤ 0.001

## Discussion

The inflammatory microenvironment of OP in human and murine lungs is still not well understood. Previous analyses have for the most part focused on OP-related lung diseases, in particular BO (Tab. 4). In these studies, similarly to our present study, TGFB1/Tgfb1, TIMP1/Timp1, COL3A1/Col3a1 and CXCL12/Cxcl12 were analyzed in human patients who suffered from BO following lung transplantation as well as in heterotopic tracheal transplantation (“BO-like”) and “IPF-like” bleomycin mouse models (Table 4) [10, 16–21]. Here, we aimed to investigate which inflammation-related factors expressed in our OP mouse model are comparable with the corresponding results in human patients. Notably, in both OP mouse and human tissues from OP patients we found comparable expression levels of pivotal genes, such as TGFB1/Tgfb1, TIMP1/Timp1, TIMP2/Timp2, COL3A1/Col3a1, CXCL12/Cxcl12, MMP2/Mmp2 and IL6/Il6. These results allow us to conclude that the transgenic CCL2 mouse model not only shows pathogenomic, molecular and morphological features of human OP but also exhibits a similar inflammatory profile.

**Table 4:** Literature review of the investigated inflammation- and fibrosis-associated genes in bronchiolitis obliterans (BO), idiopathic pulmonary fibrosis (IPF) and organizing pneumonia (OP) humans and mice

References	Pulmonary disease: no. of cases/controls	Materials	Investigated parameters	Methods
*Human*				
Magnan et al. [16]	BO: 6/7	BAL (TBB) from lung transplant recipients	*TGFB1*, *IL*-*6*, TGFB2	Immunoassay for cytokine determination Immunohistochemistry
Taghavi et al. [19]	BO: 38/34	BAL (TBB) from lung transplant patients	*MMP2*, MMP8, MMP9, *TIMP1*, *TIMP2*, BAL cell count	ELISA
Ramirez et al. [22]	BO: 13/21	BALF from lung transplant recipients	IL-1b, IL-2, IL-4, IL-5, *IL*-*6*, IL-10, IL-12, IFN-γ, GM-CSF, TNF-α, *TGFB1*, fibronectin gene expression, gelatinolytic activity	Multiplex Bead Immunoassay ELISA Gelatin zymography
Jonigk et al. [10]	BO: 12/12	Explanted lung allografts	*BMP4*, BMPR2, LOX, MMP14, PLAU, CCL5, *THBS1*, *TIMP1*, *TGFB1*, *COL3A1*, *TIMP2*, TGFBR2, BMP2, PLA1, EDN1, COL1A2, COL4A1	RNA microarray (45 target genes)
*Mouse*				
Majeski et al. [23]	OP:	Reovirus 1/L induction of intraluminal fibrosis	*Il*-*6*, *Ccl2*	RiboQuant multiprobe ribonuclease protection assay ELISA
Phillips et al. [18]	IPF: 3–6 mice for each treatment group (bleomycin/saline)	Bleomycin mouse model	*Cxcr4*, *Cxcl12*, Col1, CD45, CD34, α-SMA, Col3	FACS, ELISA, qRT-PCR, Immunohistochemistry
Chen et al. [17]	BO: 4/4	Heterotopic tracheal transplantation from TIMP-1 null (-/-) mutation and wild-type (TIMP-1 +/+) mice	Βeta-actin, Mmp3, Mmp7, Mmp9, Mmp12, *Timp1*, *Timp2*, Timp3, Timp4	Immunohistochemistry qRT-PCR
Xu [24]	BO: 6/6	Heterotopic tracheal transplantation	*Cxcr4*	Immunohistochemistry Immunofluorescence Western blot
Vittal et al. [20]	IPF:	Bleomycin mouse model	*Tgfb1*, *FAK* (*PTK2*), PKB/Akt	qRT-PCR Western blot Immunohistochemistry
Shi et al. [21]	IPF:	Bleomycin mouse model	*Tgfb1*, *Smad3*	Immunohistochemistry

Significantly regulated genes are highlighted in italics

*ACTB* beta-actin, *BAL* bronchoalveolar lavage, *BMP* bone morphogenetic protein, *BMPR* bone morphogenetic protein receptor, *CCL* CC chemokine ligand, *CD40LG* CD40 ligand, *Cxcr4* chemokine (C-X-C motif) receptor 4, *Cxcl12* chemokine (C-X-C motif) ligand 12, *ECP* eosinophilic cationic protein, *FAK (PTK)* focal adhesion kinase (protein tyrosine kinase), *FasL* Fas ligand, *FGF1* fibroblast growth factor 1, *FOXP3* forkhead box P3, *GM*-*CSF* granulocyte macrophage colony-stimulating factor, *HLA* human leukocyte antigen, *IFN* interferon, *IL* interleukin, *IP10* interferon gamma-induced protein 10, *LTA* lymphotoxin-α, *LOX* lysyl oxidase, *MCP1* monocyte chemotactic protein, *MIP* macrophage inflammatory protein, *MIG (CXCL9)* monokine induced by gamma interferon, *MMP* matrix metalloproteinase, *MPO* myeloperoxidase, *NG* neutrophilic granulocytes, *PDGFB* platelet-derived growth factor subunit B, *PLAU* plasminogen activator (urokinase), *RPL13A* 60S ribosomal protein L13a, *sCD30* soluble CD30, *SLPI* secretory leukocyte protease inhibitor, *Smad* Sma and Mad-related protein family members, *sRAGE* soluble receptor for advanced glycation end products, *TGF* transforming growth factor, *THBS1* thrombospondin-1, *TIMP1* tissue inhibitor of matrix metalloproteinases 1, *TNFα* tumor necrosis factor, alpha, *TNFSF7* TNF ligand superfamily member 7, *VEGF* vascular endothelial growth factor, *YKL*-*40* chitinase-like glycoprotein

It has previously been shown that CCL2 Tg mice are prone to develop fibrotic pulmonary lesions in terms of OP, following bacterial infection [9]. In the current study, we showed that human CCL2 overexpression adds to the endogenous murine Ccl2 expression in CCL2 Tg mice. CCL2 is a major monocyte chemoattractant produced by macrophages, lymphocytes, basophils, epithelial cells, endothelial cells and (myo)fibroblasts [25], and is involved in the pathogenesis of a variety of inflammatory lung diseases [4, 26]. In the context of bacterial pneumonia, Ccl2 knockout mice have been shown to exhibit a decreased monocytic immune response [27]. In our previous analyses, we found that CCL2 Tg mice exhibited an improved pneumococcal clearance after infection with *S. pneumoniae*, but at the same time developed OP lesions. Specific blockade of the CCL2 receptor CCR2 with function-blocking antibody MC21 resulted in both reduced bacterial clearance and abrogation of OP manifestations [9]. Therefore, combined CCL2/Ccl2 expression in lungs of transgenic mice is most likely associated with manifestation of OP lesions in the small airways and adjacent alveoli along with a complementary and multifocal accumulation of inflammatory cells.

The fact that the CCL2 transgenic animals without *S. pneumonia* infection did not developed OP, points to the importance of the infections in the disease’s development. On the other hand, infected CCL2 transgenic animals developing OP showed significantly higher expression of CCL2 than those infected CCL2 animals that did not develop OP. Hence, our data suggested a direct relationship between *S. pneumonia* infection, high expression of CCL2 and the development of OP.

In OP lesions, increased expression of proinflammatory mediators indicates that leukocytes and stromal cells generate a specific microenvironment, which in turn helps to recruit more cells to sites of inflammation. We could demonstrate these compartment-specific differences in gene expression by analyzing and comparing laser-microdissected OP lesions and adjacent non-remodeled areas within the same animals. We found that the molecular profile in murine and human OP lesions was characterized by increased expression of proinflammatory signaling factors (Cxcl12/Cxcr4 in mice, CXCL12 in humans) and profibrotic mediators (murine/human Tgfb1/TGFB1 and Timps/TIMPs). Cxcl12 is produced and secreted by fibroblasts, while its complementary receptor Cxcr4 is expressed by leukocytes [10, 11]. In a murine heterotopic tracheal transplant model with tracheal obliteration by granulation tissue and other murine bleomycin-associated fibrosis models, Cxcl12-inhibition resulted in an attenuation of fibroblastic trafficking/migration, which underlines the importance of the Cxcl12/Cxcr4 axis for generating a profibrotic pulmonary microenvironment [18, 28, 29]. Profibrotic Thbs1/THBS1, Smads/SMADs and Ptk2/PTK2 were expressed in OP lesions and these factors can, although not upregulated on the transcript level, contribute to an inflammatory microenvironment. In human patients with progressive pulmonary fibrosis, TGFB1-associated expression of fibroblastic PTK2 has been reported [20, 30] and experiments in a bleomycin-induced pulmonary fibrosis mouse model indicated that this kinase is related to fibroblastic proliferation, activation and collagen production [31].

Tgfb1 as well as Il6 are cytokines which can be produced both by leukocytes and stroma cells and both can induce an aberrant wound healing response resulting in (over)activation, migration and proliferation of (myo)fibroblasts [32–34]. In particular, Tgfb1 also induces the differentiation of fibroblast into myofibroblasts, which promotes ECM production and collagen accumulation [10, 32, 34]. Smad3 is linked to Tgfb1 signaling while Smad1 is associated with Bmp signaling pathways [35–38]. In contrast to Tgfb1, downstream Smad1 and Smad3 were not increased in murine OP lesions and both Bmp4 and its receptor Bmpr1b, were downregulated. Similar to the regulation in mice, TGFB1 was increased in human OP lesions—but in contrast to the murine setting, these factors were deregulated in an inverse fashion with increased SMADs and BMP4/BMPR1B. Bmp4 is known to inhibit fibroblastic activation and proliferation on its own, while together with Tgfb1, it further promotes differentiation of lung fibroblasts into myofibroblasts [32, 34, 39]. Therefore, myofibroblastic activation in human OP is most likely mediated by the TGFB1/SMAD3 axis, as well as by BMP4/SMAD1 signaling, while in mice an OP-restricted decrease of murine Bmp4/Bmpr1b could contribute to aggregation of myofibroblasts within the lesions.

In bronchoalveolar lavage fluids from human patients with OP, IL6 was shown to be increased [40], while in other studies in lung-transplanted humans, alveolar macrophage-derived IL6 was increased at the time of onset of acute rejection, but not in patients who developed chronic lung allograft dysfunction (CLAD) [16]. In our analyses of mice and humans, the local expression levels of IL6 were comparable in remodeled and non-remodeled segments of the lung. This indicates an unspecific reaction to bacterial infection, suggesting that IL6 expression was in response to infection, but not in an OP-specific manner, which affects all compartments of the lung. However, the major driver of inflammatory processes within the murine and human OP lesions was not Il6/IL6 but Tgfb1/TGFB1. Tgfb1 is secreted in the form of an inactive complex which becomes activated by latency-associated protein (LAP) and Tgfb1/LMP dissociation, which in turn can be induced by several factors, such as thrombospondins (THBS) and Mmps [35, 41]. Thbs1, as well as Mmp inhibitors Timp1 and Timp2 are in turn typically associated with Tgfb1-induced fibrotic tissue remodeling [32, 41]. Our analyses revealed that Timps/TIMPs were increased in murine and human OP lesions alike, which was also paralleled by an increased expression of collagens, thus resulting in accumulation of ECM. Matrix cleavage enzyme Mmp2 was significantly upregulated in human and to a lesser extent murine OP. Since Mmp2/MMP2 can be inhibited by Timps/TIMPs, accumulation of ECM components is increased in both systems. Furthermore, the matricellular profibrotic factor Thbs1 was upregulated in murine—but not in human—OP lesions. In human BO lesions, THBS1 has been shown to be overexpressed, which might indicate a different mechanism of THBS1-mediated fibrosis in human OP and BO [10].

## Conclusions

In summary, lung bacterial infections in the presence of high intra-alveolar levels of CCL2 rendered mice susceptible to developing OP lesions. Compartment-specific analysis of laser-microdissected lung tissue in a mouse model of infection-induced OP revealed that, complementary to histomorphological changes, OP lesions show a characteristic proinflammatory molecular profile in mice as well as in humans with OP. The OP microenvironment typically shows higher levels of Tgfb1 and Cxcl12/Cxcr4 signaling factors, profibrotic Thbs1 and inhibitors of collagen degradation Timp1/Timp2. Comparative gene expression analysis in human and murine OP lesions displayed in part similar expression levels, notably of TGFB1/Tgfb1, TIMP1/Timp1, TIMP2/Timp2, COL3A1/Col3a1, CXCL12/Cxcl12, MMP2/Mmp2 and IL6/Il6. Therefore, the currently employed humanized mouse model of lung-specific CCL2 overexpression shows pathogenomic morphological and molecular features of human OP, making this animal model suitable for further investigation of fibrotic pulmonary remodeling, particularly of OP, in regard to pathogenesis and potential therapeutic strategies.

## Abbreviations

BMPs: bone morphogenetic proteins
BMPR1B: bone morphogenetic protein receptor type-1B
BO: bronchiolitis obliterans
BOOP: bronchiolitis obliterans organizing pneumonia
CCL2: C-C chemokine ligand 2
CLAD: chronic lung allograft dysfunction
COL3A1: collagen type III, alpha 1
COP: cryptogenic organizing pneumonia
CXCL12: chemokine (C-X-C motif) ligand 12
CXCR4: chemokine (C-X-C motif) receptor 4
ECM: extracellular matrix
FFPE: formalin-fixed and paraffin-embedded
IL6: Interleukin 6
IPF: idiopathic pulmonary fibrosis
MMPs: matrix metalloproteinases
OAR: obliterative airway remodeling
OP: organizing pneumonia
POLR2A: DNA-directed RNA polymerase II subunit RPB1
PTK2: protein tyrosine kinase 2
SMADs: Sma and Mad-related protein family members
SOP: secondary organizing pneumonia
TGFB1: transforming growth factor beta 1
THBS1: thrombospondin 1
TIMPs: tissue inhibitors of matrix metalloproteinases

## References

[1] American Thoracic Society (2002). European Respiratory Society: American Thoracic Society/European Respiratory Society International Multidisciplinary Consensus Classification of the Idiopathic Interstitial Pneumonias. This joint statement of the American Thoracic Society (ATS), and the European Respiratory Society (ERS) was adopted by the ATS board of directors, June 2001 and by the ERS Executive Committee, June 2001. Am J Respir Crit Care Med.

[2] Hamer OW, Silva CI, Muller NL (2008). Cryptogenic organizing pneumonia: typical and atypical imaging features on computed tomography. Rofo.

[3] Roberton BJ, Hansell DM (2011). Organizing pneumonia: a kaleidoscope of concepts and morphologies. Eur Radiol.

[4] Cordier JF (2006). Cryptogenic organising pneumonia. Eur Respir J.

[5] Beardsley B, Rassl D (2013). Fibrosing organising pneumonia. J Clin Pathol.

[6] Drakopanagiotakis F, Polychronopoulos V, Judson MA (2008). Organizing pneumonia. Am J Med Sci.

[7] Ranzani OT, Parra ER, de Morais Fernezlian S, Capelozzi VL (2007). Intraluminal plugs in idiopathic and secondary organizing pneumonia: repair or remodelling?. Histopathology.

[8] Jungraithmayr W, Jang JH, Schrepfer S, Inci I, Weder W (2013). Small animal models of experimental obliterative bronchiolitis. Am J Respir Cell Mol Biol.

[9] Winter C, Taut K, Srivastava M, Langer F, Mack M, Briles DE, Paton JC, Maus R, Welte T, Gunn MD, Maus UA (2007). Lung-specific overexpression of CC chemokine ligand (CCL) 2 enhances the host defense to *Streptococcus pneumoniae* infection in mice: role of the CCL2–CCR2 axis. J Immunol.

[10] Jonigk D, Merk M, Hussein K, Maegel L, Theophile K, Muth M, Lehmann U, Bockmeyer CL, Mengel M, Gottlieb J, Welte T, Haverich A, Golpon H, Kreipe H, Laenger F (2011). Obliterative airway remodeling: molecular evidence for shared pathways in transplanted and native lungs. Am J Pathol.

[11] Jonigk D, Theophile K, Hussein K, Bock O, Lehmann U, Bockmeyer CL, Gottlieb J, Fischer S, Simon A, Welte T, Maegel L, Kreipe H, Laenger F (2010). Obliterative airway remodelling in transplanted and non-transplanted lungs. Virchows Arch.

[12] Gunn MD, Nelken NA, Liao X, Williams LT (1997). Monocyte chemoattractant protein-1 is sufficient for the chemotaxis of monocytes and lymphocytes in transgenic mice but requires an additional stimulus for inflammatory activation. J Immunol.

[13] Peters K, Werner S, Liao X, Wert S, Whitsett J, Williams L (1994). Targeted expression of a dominant negative FGF receptor blocks branching morphogenesis and epithelial differentiation of the mouse lung. EMBO J.

[14] Lehmann U, Kreipe H (2001). Real-time PCR analysis of DNA and RNA extracted from formalin-fixed and paraffin-embedded biopsies. Methods.

[15] Theophile K, Jonigk D, Kreipe H, Bock O (2008). Amplification of mRNA from laser-microdissected single or clustered cells in formalin-fixed and paraffin-embedded tissues for application in quantitative real-time PCR. Diagn Mol Pathol.

[16] Magnan A, Mege JL, Escallier JC, Brisse J, Capo C, Reynaud M, Thomas P, Meric B, Garbe L, Badier M, Viard L, Bongrand P, Giudicelli R, Metras D, Fuentes P, Vervloet D, Noirclerc M (1996). Balance between alveolar macrophage IL-6 and TGF-beta in lung-transplant recipients. Marseille and Montreal Lung Transplantation Group. Am J Respir Crit Care Med.

[17] Chen P, Farivar AS, Mulligan MS, Madtes DK (2006). Tissue inhibitor of metalloproteinase-1 deficiency abrogates obliterative airway disease after heterotopic tracheal transplantation. Am J Respir Cell Mol Biol.

[18] Phillips RJ, Burdick MD, Hong K, Lutz MA, Murray LA, Xue YY, Belperio JA, Keane MP, Strieter RM (2004). Circulating fibrocytes traffic to the lungs in response to CXCL12 and mediate fibrosis. J Clin Invest.

[19] Taghavi S, Krenn K, Jaksch P, Klepetko W, Aharinejad S (2005). Broncho-alveolar lavage matrix metalloproteases as a sensitive measure of bronchiolitis obliterans. Am J Transplant.

[20] Vittal R, Horowitz JC, Moore BB, Zhang H, Martinez FJ, Toews GB, Standiford TJ, Thannickal VJ (2005). Modulation of prosurvival signaling in fibroblasts by a protein kinase inhibitor protects against fibrotic tissue injury. Am J Pathol.

[21] Shi K, Jiang J, Ma T, Xie J, Duan L, Chen R, Song P, Yu Z, Liu C, Zhu Q, Zheng J (2014). Pathogenesis pathways of idiopathic pulmonary fibrosis in bleomycin-induced lung injury model in mice. Respir Physiol Neurobiol.

[22] Ramirez AM, Nunley DR, Rojas M, Roman J (2008). Activation of tissue remodeling precedes obliterative bronchiolitis in lung transplant recipients. Biomark Insights.

[23] Majeski EI, Paintlia MK, Lopez AD, Harley RA, London SD, London L (2003). Respiratory reovirus 1/L induction of intraluminal fibrosis, a model of bronchiolitis obliterans organizing pneumonia, is dependent on T lymphocytes. Am J Pathol.

[24] Xu J, Torres E, Mora AL, Shim H, Ramirez A, Neujahr D, Brigham KL, Rojas M (2008). Attenuation of obliterative bronchiolitis by a CXCR4 antagonist in the murine heterotopic tracheal transplant model. J Heart Lung Transplant.

[25] Rose CE, Sung SS, Fu SM (2003). Significant involvement of CCL2 (MCP-1) in inflammatory disorders of the lung. Microcirculation.

[26] Kennedy VE, Todd JL, Palmer SM (2013). Bronchoalveolar lavage as a tool to predict, diagnose and understand bronchiolitis obliterans syndrome. Am J Transplant.

[27] Winter C, Herbold W, Maus R, Langer F, Briles DE, Paton JC, Welte T, Maus UA (2009). Important role for CC chemokine ligand 2-dependent lung mononuclear phagocyte recruitment to inhibit sepsis in mice infected with *Streptococcus pneumoniae*. J Immunol.

[28] Harris DA, Zhao Y, LaPar DJ, Emaminia A, Steidle JF, Stoler M, Linden J, Kron IL, Lau CL (2013). Inhibiting CXCL12 blocks fibrocyte migration and differentiation and attenuates bronchiolitis obliterans in a murine heterotopic tracheal transplant model. J Thorac Cardiovasc Surg.

[29] Song JS, Kang CM, Kang HH, Yoon HK, Kim YK, Kim KH, Moon HS, Park SH (2010). Inhibitory effect of CXC chemokine receptor 4 antagonist AMD3100 on bleomycin induced murine pulmonary fibrosis. Exp Mol Med.

[30] Lagares D, Busnadiego O, Garcia-Fernandez RA, Kapoor M, Liu S, Carter DE, Abraham D, Shi-Wen X, Carreira P, Fontaine BA, Shea BS, Tager AM, Leask A, Lamas S, Rodriguez-Pascual F (2012). Inhibition of focal adhesion kinase prevents experimental lung fibrosis and myofibroblast formation. Arthritis Rheum.

[31] Kinoshita K, Aono Y, Azuma M, Kishi J, Takezaki A, Kishi M, Makino H, Okazaki H, Uehara H, Izumi K, Sone S, Nishioka Y (2013). Antifibrotic effects of focal adhesion kinase inhibitor in bleomycin-induced pulmonary fibrosis in mice. Am J Respir Cell Mol Biol.

[32] Fernandez IE, Eickelberg O (2012). The impact of TGF-beta on lung fibrosis: from targeting to biomarkers. Proc Am Thorac Soc.

[33] Liu X, Das AM, Seideman J, Griswold D, Afuh CN, Kobayashi T, Abe S, Fang Q, Hashimoto M, Kim H, Wang X, Shen L, Kawasaki S, Rennard SI (2007). The CC chemokine ligand 2 (CCL2) mediates fibroblast survival through IL-6. Am J Respir Cell Mol Biol.

[34] Yan Z, Kui Z, Ping Z (2014). Reviews and prospectives of signaling pathway analysis in idiopathic pulmonary fibrosis. Autoimmun Rev.

[35] Borthwick LA, Wynn TA, Fisher AJ (2013). Cytokine mediated tissue fibrosis. Biochim Biophys Acta.

[36] Miyazono K, Kamiya Y, Morikawa M (2010). Bone morphogenetic protein receptors and signal transduction. J Biochem.

[37] Warburton D, Shi W, Xu B (2013). TGF-beta-Smad3 signaling in emphysema and pulmonary fibrosis: an epigenetic aberration of normal development?. Am J Physiol Lung Cell Mol Physiol.

[38] Sahin H, Wasmuth HE (2013). Chemokines in tissue fibrosis. Biochim Biophys Acta.

[39] Pegorier S, Campbell GA, Kay AB, Lloyd CM (2010). Bone morphogenetic protein (BMP)-4 and BMP-7 regulate differentially transforming growth factor (TGF)-beta1 in normal human lung fibroblasts (NHLF). Respir Res.

[40] Cai M, Bonella F, Dai H, Sarria R, Guzman J, Costabel U (2013). Macrolides inhibit cytokine production by alveolar macrophages in bronchiolitis obliterans organizing pneumonia. Immunobiology.

[41] Ide M, Ishii H, Mukae H, Iwata A, Sakamoto N, Kadota J, Kohno S (2008). High serum levels of thrombospondin-1 in patients with idiopathic interstitial pneumonia. Respir Med.

